# The COPD multi-dimensional phenotype: A new classification from the STORICO Italian observational study

**DOI:** 10.1371/journal.pone.0221889

**Published:** 2019-09-13

**Authors:** Raffaele Antonelli Incalzi, Giorgio Walter Canonica, Nicola Scichilone, Sara Rizzoli, Lucia Simoni, Francesco Blasi, on behalf of STORICO study group

**Affiliations:** 1 University Biomedical Campus of Rome, Rome, Italy; 2 Personalized Medicine Asthma & Allergy Clinic Humanitas University Humanitas research Hospital, Rozzano, Milan, Italy; 3 DIBIMIS, University of Palermo, Piazza delle Cliniche, Palermo, Italy; 4 Medineos Observational Research, Modena, ltaly; 5 Department of Pathophysiology and Transplantation, University of Milan, Internal Medicine Department, Respiratory Unit and Cystic Fibrosis Adult Center Fondazione IRCCS Cà Granda Maggiore Hospital, Milan, Italy; National and Kapodistrian University of Athens, GREECE

## Abstract

**Background:**

This paper is aimed to (i) develop an innovative classification of COPD, *multi-dimensional phenotype*, based on a multidimensional assessment; (ii) describe the identified *multi-dimensional phenotypes*.

**Methods:**

An exploratory factor analysis to identify the main classificatory variables and, then, a cluster analysis based on these variables were run to classify the COPD-diagnosed 514 patients enrolled in the STORICO (trial registration number: NCT03105999) study into *multi-dimensional phenotypes*.

**Results:**

The circadian rhythm of symptoms and health-related quality of life, but neither comorbidity nor respiratory function, qualified as primary classificatory variables. Five *multidimensional phenotypes* were identified: the MILD COPD characterized by no night-time symptoms and the best health status in terms of quality of life, quality of sleep, level of depression and anxiety, the MILD EMPHYSEMATOUS with prevalent dyspnea in the early-morning and day-time, the SEVERE BRONCHITIC with nocturnal and diurnal cough and phlegm, the SEVERE EMPHYSEMATOUS with nocturnal and diurnal dyspnea and the SEVERE MIXED COPD distinguished by higher frequency of symptoms during 24h and worst quality of life, of sleep and highest levels of depression and anxiety.

**Conclusions:**

Our results showed that properly collected respiratory symptoms play a primary classificatory role of COPD patients. The longitudinal observation will disclose the discriminative and prognostic potential of the proposed multidimensional phenotype.

**Trial registration:**

Trial registration number: NCT03105999, date of registration: 10th April 2017.

## Introduction

Chronic Obstructive Pulmonary Disease (COPD) is an umbrella definition encompassing a variety of clinical and pathophysiological conditions. Selected clinical phenotypes, namely the bronchitic, the emphysematous and the asthma-like, have been repeatedly recognized, but disease pattern not always univocally conforms to one of them [1]. Indeed, the clinical presentation frequently resembles a mixture of the classical phenotypes. Furthermore, comorbidity and age itself largely shape the clinical and health status of COPD patients. Indeed, a senescence-associated secretory phenotype has been identified and might affect the clinical expression of COPD in the elderly [2]. Unfortunately, large pharmacological randomized clinical trials (RCTs) could not shed light on phenotypic variability due to the stringent selecting criteria. Further complicating this issue is the lack of attention to the circadian rhythm of respiratory symptoms in RCTs. On the other hand, several observational studies point out at circadian symptom variability as a correlate of disease severity [3] or a distinctive clinical trait [4] or a correlate of derangement of selected health status dimensions [5]. Furthermore, nocturnal symptoms are reported by the majority of COPD patients [6] and dramatically increased with age in a COPD population over 65 [7]. However, circadian rhythm variability in COPD is only marginally and partially explored in selected RCT with the only intent of providing 24 hours pharmacological coverage and, then, symptoms control. Thus, how circadian rhythm of symptoms affects the clinical patterns and therapeutic needs of COPD has not been the object of interest. Paradoxically, the growing interest in comorbidity of COPD and its role as a determinant of health status seems to overcome the research on the respiratory dimension of COPD.

The STORICO (STudio Osservazionale sulla caratteRizzazione dei sIntomi delle 24 ore nei pazienti con broncopneumopatia cronica ostruttiva, *Observational study on characterization of 24-h symptoms in patients with COPD*) study assessed the association between circadian rhythm of symptoms and several measures of health status [8]. Thus, it represents the ideal framework to verify whether an in depth characterization of symptoms may allow define clinical variants of COPD with distinctive classificatory and discriminatory properties. Thus, the present paper is aimed to:

- develop an innovative classification of COPD based on a multidimensional assessment (named *multi-dimensional phenotype*) which takes into consideration primarily circadian rhythm of symptoms, but also demographic characteristics, health related quality of life, respiratory function and comorbidities.
- describe the patients belonging to the so-identified *multi-dimensional phenotypes* with respect to body mass index, respiratory parameters, previous exacerbations, level of physical activity, quality of sleep, level of depression and anxiety and ongoing COPD pharmacological therapies.

The final objective of such an attempt is to investigate the discriminatory properties and potential clinical implications of this alternative classificatory method.

## Materials and methods

### Study subjects

The STORICO study (trial registration number: NCT03105999) enrolled subjects aged ≥50, current/ex-smokers, with a diagnosis of stable COPD according to the GOLD 2014. The study was approved by the ethical committee of the coordinating center (Fondazione Toscana G. Monasterio Pisa, Italy) and was conducted in accordance with the Declaration of Helsinki and the Good Clinical Practices guidelines for observational studies, complying with all requirements of local regulations. Patients provided written, informed consent before study participation. Subjects included, study design and methodology of the STORICO study are extensively described elsewhere [8].

Patients with available information about early-morning, day- and night-time COPD symptoms and clinical phenotype at enrollment were considered evaluable for analyses at baseline; among these, the ones with available variables for (factor and cluster) analyses (below detailed in Methods paragraph) were analyzed in this paper.

### Study design

STORICO is an Italian observational cohort multicentre currently ongoing study conducted in 40 pneumology centers. The study lasted from February 2016 (first subject first visit) to June 2018 (last subject last visit); three visits were planned (baseline, 6- and 12-months follow up).

### Methods

At enrollment patients completed the *Night-time*, *Morning and Day-time Symptoms of COPD questionnaire* [5] (hereafter named “symptoms questionnaire”) covering the frequency and severity of COPD symptoms (breathlessness, coughing, bringing up phlegm or mucus, chest tightness, chest congestion and wheezing) during each part of the day (night-time, early- and day-time). Linguistic validation of the questionnaire in Italian was performed by the authors to ensure accurate translation and a clear understanding of the questionnaire itself [5].

Health Related Quality of Life was evaluated by the *St*. *George’s Respiratory Questionnaire (SGRQ)*, in its Symptoms, Activity component and Impact scores [9–10] ranging between 0 (no impairment) and 100 (highest impairment), lower scores corresponding to better health.

Anxiety and depression levels were evaluated through the *Hospital Anxiety and Depression Scale (HADS)* [11], a total score (ranging 0–42) and anxiety and depression subscales scores (ranging 0–21) were computed, with higher scores indicating more distress.

The impact on sleep due to respiratory disease was assessed with the total score of *COPD and Asthma Sleep Impact Scale (CASIS)* [12], ranging 0–100, with higher scores indicating greater sleep impairment.

Physical activity was assessed by means of the categorical score (low, medium, high physical activity) of the *International Physical Activity Questionnaire* (IPAQ) [13] on patients aged 15–69 years. Spirometry was performed according to the recommendations of the American Thoracic Society (ATS) and the European Respiratory Society (ERS) and lung function measurements were done with patients either standing or sitting with the nose clipped after at least 10 minutes rest [14].

Presence of relevant comorbidities according to clinical judgment, body mass index (BMI), spirometry functional assessment and occurrence of exacerbations in the 5 years before baseline were recorded too as far as ongoing pharmacological therapies for COPD (long-acting beta-agonists (LABA), long-acting muscarinic antagonist (LAMA), inhaled corticosteroid (ICS) and any combination, Others).

### Analysis

In order to identify the multi-dimensional phenotypes (*m-phenotypes*), a multi-step approach was followed.

#### STEP 1—Exploratory factor analysis

An exploratory factor analysis (EFA) was performed to find independent latent constructs (factors), not directly measurable and influencing responses on observed variables. EFA is a variable reduction technique which does not impose any preconceived structure on the outcome [15] and the observed variables included in the model are a linear combination of the underlying factors.

The following items of the symptoms questionnaire were included in the factor analysis: presence/absence of feeling short of breath or breathless (items 18a, 26a, 7a), cough (items 18b, 26b, 7b), bringing up phlegm or mucus (items 18c, 26c, 7c), each symptom evaluated during the 24-hours (i.e. in the early-morning, day-time and night-time). The presence of the symptom was coded as 1 and the absence as 0.

The value of the FEV1% of the predicted, of (symptoms, activity, impact) SGRQ scores, demographic variables (age as continuous variable and gender coded as 1 for males and 2 for females) and presence/absence of relevant comorbidities (cardiac ischemic disease, arterial hypertension, heart failure, atrial fibrillation, diabetes, osteoporosis, depression, kidney insufficiency) were also included in the model (presence was coded as 1 and absence as 0). Orthogonal VARIMAX rotation was applied; factors having an eigenvalue > 1.0 [16–17] and individual variables with higher-than-0.5 loadings on retained factors were retained [18].

#### STEP 2 –Definition of classificatory variables

STEP 1 brought to identification of *n* factors. Then, the variables included in the *n*-th factor were evaluated and, whenever possible, combined into a new classificatory variable (a classificatory variable for each factor was defined).

#### STEP 3 –Cluster analysis

A cluster analysis with classificatory variables mentioned at STEP 2 as input was performed. Average linkage was chosen as clustering method and average distance between clusters equal to 0.70 was taken as reference to cut the dendrogram. Quality of obtained clusters was evaluated by means of Semipartial R-squared (i.e. the loss of homogeneity due to combining two clusters to form a new cluster) and R-square (i.e. the proportion of variance accounted for by the clusters).

Once identified, the different m-phenotypes were described with respect to BMI, spirometric parameters, number of previous exacerbations, quality of sleep, level of physical activity, depression and anxiety and therapies for COPD at enrollment by means of median, 25^th^ and 75^th^ percentile for quantitative variables and absolute/relative frequency for categorical ones within each class of m-phenotype. Moreover, although SGRQ scores were included in EFA, SGRQ scores of m-phenotypes were compared to provide a comprehensive description of health status.

Analysis of variance and Kruskal–Wallis test by ranks (on means and medians respectively) and Fisher exact test (for categorical variables) were used to compare variables vs m-phenotype (variable in 5 classes). Upon statistical significance of these tests, Mann-Whitney test on medians and Chi-square or Fisher exact tests were then applied to compare variables among specific pairs of m-phenotypes. Alpha (with Bonferroni correction) was set to 0.0004 considering the total number of performed tests.

Statistical analysis was performed using SAS v9.4 and Enterprise Guide v7.1.

## Results

### Evaluable patients

**Fig 1:**
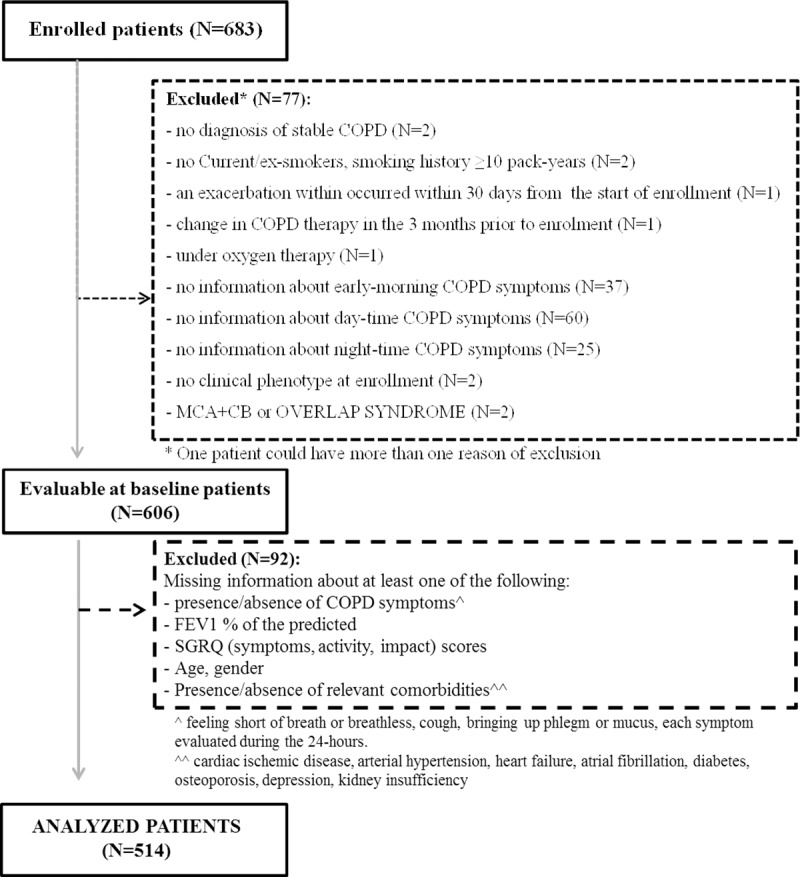
Patient disposition. The figure shows the number of patients enrolled at baseline, evaluable at baseline and analyzed and the reasons for exclusion.

Among the 683 COPD patients enrolled in the STORICO study, 606 (88.7%) subjects (age 71.4±8.2 years, 75.1% males) were deemed evaluable for the analysis at baseline; 92 subjects were then excluded because they had missing information on variables analyzed in the factor and cluster analyses. Violations causing exclusion are shown in Fig 1.

So, 514 (mean±SD age: 71.4± 8.0 years) were deemed evaluable for the analyses here described.

### Identification of m-phenotypes

#### STEP 1—Exploratory factor analysis

As results of the factors analysis, two factors accounting for 82% of the total variability were retained. In Table 1, the factor loadings of individual variables are shown. Factor 1 covers 58.6% of variability and its components are bringing up phlegm or mucus and cough at any time of the day. Factor 2, explaining 23.2% of variability, is composed by breathlessness at any time of the day and by SGRQ scores. A third factor explained a reduced amount of variability (9.5%) and no variables had a factor loading >0.5; so, it was not retained (output of factor analysis is reported in S1 Table and S1 Fig).

**Table 1:** Factor 1, 2 and 3: Factor loadings.

	Factor1	Factor2	Factor3
Bringing up phlegm or mucus (DAY-TIME)	**0.76567**	0.08402	0.06274
Bringing up phlegm or mucus (EARLY-MORNING)	**0.75702**	0.01087	0.04818
Cough (DAY-TIME)	**0.71960**	0.13606	-0.08848
Cough (EARLY-MORNING)	**0.70750**	0.12600	-0.09838
Bringing up phlegm or mucus (NIGHT-TIME)	**0.58962**	0.22837	0.10707
Cough (NIGHT-TIME)	**0.57541**	0.25280	-0.05965
Osteoporosis	-0.09562	0.03034	-0.09537
SGRQ (activity score)	0.12888	**0.79973**	0.07505
SGRQ (impacts score)	0.22687	**0.79011**	-0.01568
SGRQ (symptoms score)	**0.53305**	**0.66753**	-0.03373
Breathlessness (EARLY-MORNING)	0.08851	**0.65837**	0.00826
Breathlessness (DAY-TIME)	0.09992	**0.62001**	-0.00822
Breathlessness (NIGHT-TIME)	0.18258	**0.51578**	-0.05343
Heart failure	-0.01054	0.04432	0.02073
FEV1 of the predicted (%)	-0.02539	-0.31861	-0.06188
Age at enrollment visit (years)	-0.02229	0.08622	0.41033
Cardiac ischemic disease	0.02193	0.00187	0.33738
Arterial Hypertension	0.02618	0.00669	0.31897
Diabetes	-0.00256	0.02765	0.28320
Kidney Insufficiency	-0.03733	0.06382	0.22236
Atrial fibrillation	-0.06142	0.02546	0.20271
Depression	-0.08237	0.08699	-0.09820
Gender	-0.09778	0.07278	-0.34109

Orthogonal VARIMAX rotation applied.

Factor loadings >0.5 are in bold.

#### STEP 2 –definition of classificatory variables

The items of symptoms questionnaire contributing to each factor (shown in grey in Table 1), were combined into 2 new 3-levels classificatory variables, namely “cough and/or bringing up phlegm or mucus” and “breathlessness”. The levels of them had increasing severity and were defined based on the frequency of occurrence of the symptoms: (i) never during 24 hours, (ii) in the early-morning and/or day-time, but never in night and (iii) in night and in the early-morning and/or day-time. The distribution of patients according to classificatory variables is shown in Table 2.

**Table 2:** Distribution of patients according to classificatory variables.

Classificatory variables (N, %)	N = 514			
Cough and/or bringing up phlegm or mucus				
never during 24 hours	127 (24.7)			
in the early-morning and/or day-time, but never in night	175 (34.1)			
in night and in the early-morning and/or day-time	212 (41.2)			
		SGRQ scores Median (25^th^ - 75^th^ percentile)		
Breathlessness		symptoms	activity	impacts
never during 24 hours	187 (36.4)	24.4 (13.1–41.3)	35.8 (23.3–47.7)	10.3 (5.5–17.8)
in the early-morning and/or day-time, but never in night	224 (43.6)	45.2 (35.0–57.2)	53.5 (41.3–64.3)	19.5 (12.5–33.8)
in night and in the early-morning and/or day-time	103 (20.0)	61.3 (50.6–75.3)	66.2 (55.1–79.5)	36.9 (21.8–57.1)

Percentages computed out of non-missing responses.

As SGRQ scores were retained in the second factor, they were described too within the levels of “breathlessness” classificatory variable (Table 2). The (symptoms, activity, impacts) scores significantly increased with level of severity of “breathlessness” (p-value < .0001 for scores in all pairwise comparisons among “breathlessness” categories).

#### STEP 3 –cluster analysis

The cluster analysis run on previous mentioned classificatory variables brought to five well defined clusters (Semipartial R-squared = 0.0260, R-square = 0.828; dendrogram is shown in S2 Table and S2 Fig). Such clusters are thereafter called *m-phenotypes* and they are below described.

### M-phenotypes and circadian rhythm of symptoms

**Fig 2:**
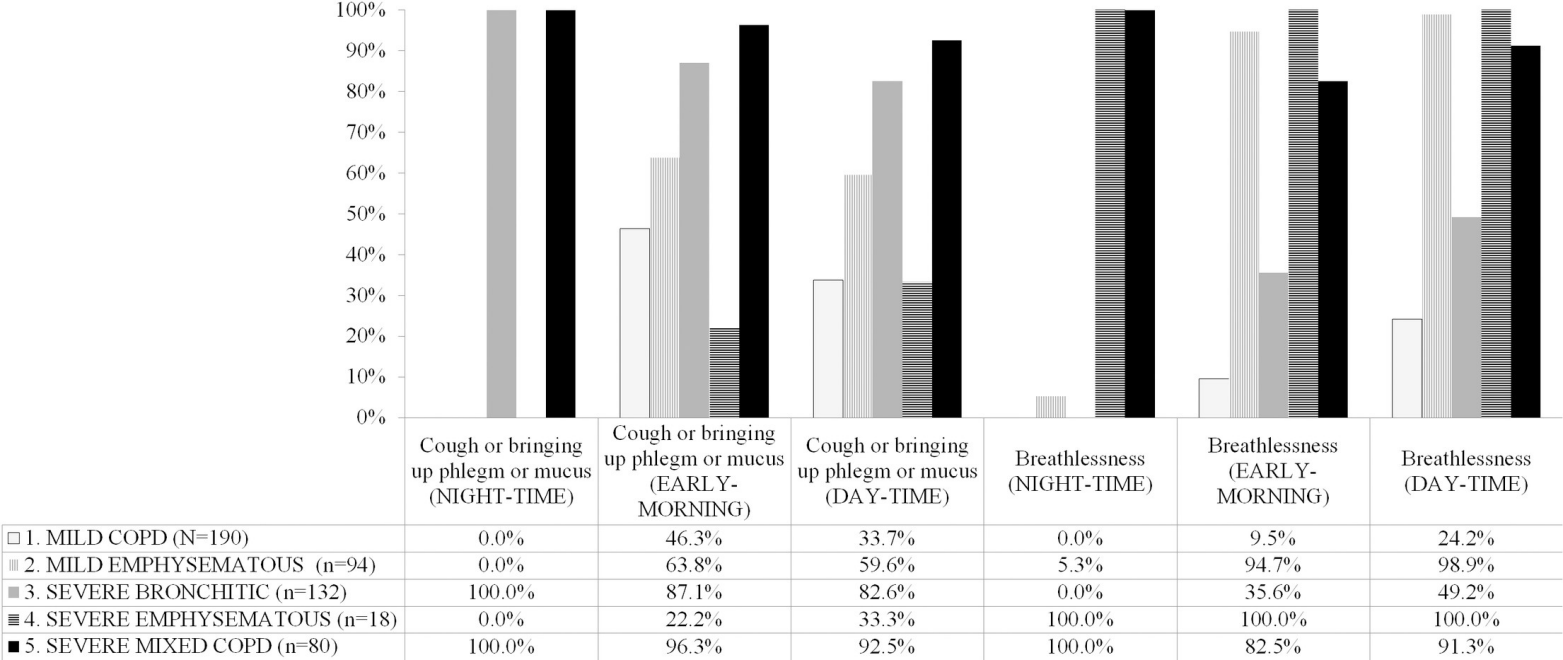
Circadian rhythm of multidimensional phenotypes. The figure shows the circadian rhythm of symptoms in the 5 multidimensional phenotypes. Percentages of symptoms during night-time, early-morning, day-time were calculated within m-phenotypes considering the patients suffering from the symptoms. A patient could have multiple symptoms. MILD COPD: No night-time symptoms. MILD EMPHYSEMATOUS: Prevalent dyspnea in the early-morning and day-time, but not by night. SEVERE BRONCHITIC: Nocturnal and diurnal cough and phlegm, no nocturnal dyspnea. SEVERE EMPHYSEMATOUS: Nocturnal and diurnal dyspnea. SEVERE MIXED COPD: Higher frequency of symptoms during 24h.

As shown in Fig 2 the m-phenotypes were characterized with regard to the circadian rhythm of symptoms as follows:

1. **No night-time symptoms (MILD COPD), n = 190.** In this m-phenotype, 88 (46.3%) and 64 (33.7%) patients presented cough and/or bringing up phlegm or mucus in the early-morning and day-time, respectively. Breathlessness was reported by 18 (9.5%) and 46 (24.2%) patients in the early-morning and day-time, respectively. Patients belonging to this phenotype have no night symptoms.
2. **Prevalent dyspnea in the early-morning and day-time, not by night (MILD EMPHYSEMATOUS), n = 94.** Patients in this cluster suffered mainly from breathlessness in the early-morning (n = 89, 94.7%) and day-time (n = 93, 98.9%). Diurnal cough or bringing up phlegm or mucus was less frequent (63.8% in the early-morning, 59.6% in the day-time), and night symptoms were distinctly rare (only breathlessness in 5.3% patients).
3. **Nocturnal and diurnal cough and phlegm, no nocturnal dyspnea (SEVERE BRONCHITIC), n = 132.** These patients suffered from cough and/or bringing up phlegm or mucus, at night (n = 132, 100.0%), in the early-morning (n = 115, 87.1%) and day-time (n = 109, 82.6%). Early-morning dyspnea is present in 47 (35.6%) patients and day-time in 65 (49.2%) patients.
4. **Nocturnal and diurnal dyspnea (SEVERE EMPHYSEMATOUS), n = 18.** At variance with MILD EMPHYSEMATOUS, they presented breathlessness also during night (n = 18, 100%).
5. **Higher frequency of symptoms, both cough and breathlessness, during 24h (SEVERE MIXED COPD), n = 80.** These patients had the highest frequency of symptoms during 24 hours: 100% of them had cough and breathlessness during night, 77 (96.3%) and 66 (82.5%) had these symptoms in the early-morning and 74 (92.5%) and 73 (91.3%) in the day-time.

### M-phenotypes vs BMI, respiratory parameters and number of exacerbations

In Table 3 BMI, spirometric parameters at enrollment and previous exacerbations are described. Patients with MILD COPD had a lower median (25^th^ - 75^th^ percentile) number of exacerbations (1.0 (0.0–3.0)) than patients with SEVERE MIXED COPD (3.0 (1.0–5.0); p-value < .0001). No other statistically significant differences emerged in analyzed parameters.

**Table 3:** Multidimensional phenotype vs BMI, respiratory parameters and previous COPD exacerbations at enrollment.

	MILD COPD	MILD EMPHYSEMATOUS	SEVERE BRONCHITIC	SEVERE EMPHYSEMATOUS	SEVERE MIXED COPD
**BMI**	n = 188	n = 92	n = 132	n = 18	n = 79
	26.6 (24.1–28.7)	26.8 (24.2–29.8)	26.9 (24.3–30.1)	26.9 (23.7–30.1)	26.5 (23.5–30.1)
**N of COPD exacerbations/year** **(5 years before baseline)**					
	n = 177	n = 91	n = 118	n = 15	n = 70
	1.0 (0.0–3.0)	2.0 (0.0–3.0)	2.0 (1.0–3.0)	2.0 (1.0–5.0)	3.0 (1.0–5.0)
**FEV1 of the predicted (%)**	n = 190	n = 94	n = 132	n = 18	n = 80
	67.0 (53.0–84.0)	61.5 (52.0–75.0)	62.6 (52.5–81.0)	58.5 (50.0–71.0)	54.5 (39.5–78.9)
**RV (L)**	n = 104	n = 61	n = 70	n = 7	n = 34
	3.1 (2.5–4.0)	3.8 (3.0–5.0)	3.0 (2.5–3.9)	3.0 (2.5–4.3)	3.2 (2.4–3.8)
**TLC (L)**	n = 101	n = 61	n = 69	n = 7	n = 33
	6.4 (5.5–7.4)	6.8 (5.9–7.8)	6.1 (5.2–7.1)	6.6 (4.8–7.3)	5.9 (5.0–6.7)
**RV/TLC**	n = 103	n = 34	n = 69	n = 7	n = 34
	0.5 (0.4–0.6)	0.6 (0.5–0.7)	0.5 (0.4–0.6)	0.5 (0.4–0.6)	0.5 (0.4–0.6)
**DLCO of the predicted (%)**	n = 76 72.6 (55.5–84.0)	n = 42 67.0 (49.7–81.1)	n = 47 65.7 (45.0–81.0)	n = 8 53.5 (39.4–71.5)	n = 21 67.0 (58.0–74.0)

BMI: Body mass index; RV: Residual Volume; TLC: Total lung capacity; DLCO: diffusing capacity of the lung for carbon monoxide.

Median (25^th^ - 75^th^ percentile) were showed.

MILD COPD: No night-time symptoms.

MILD EMPHYSEMATOUS: Prevalent dyspnea in the early-morning and day-time, but not by night.

SEVERE BRONCHITIC: Nocturnal and diurnal cough and phlegm, no nocturnal dyspnea.

SEVERE EMPHYSEMATOUS: Nocturnal and diurnal dyspnea.

SEVERE MIXED COPD: Higher frequency of symptoms during 24h.

### M-phenotypes vs quality of life, quality of sleep, level of physical activity, anxiety and depression

Quality of life, as expressed by SGRQ scores, was better in MILD COPD than in patients of other m-phenotypes (p-values tests MILD COPD vs each of the other m-phenotypes < .0001) (Table 4*)*. The SEVERE MIXED COPD m-phenotype had higher SGRQ symptoms score than the other phenotypes (p-values < .0001) and higher SGRQ activity and impacts scores than in MILD EMPHYSEMATOUS or SEVERE BRONCHITIC patients (p-values < .0001).

**Table 4:** Multidimensional phenotype vs quality of life, quality of sleep, level of physical activity and anxiety and depression at enrollment.

	MILD COPD	MILD EMPHYSEMATOUS	SEVERE BRONCHITIC	SEVERE EMPHYSEMATOUS	SEVERE MIXED COPD
*N*	*190*	*94*	*132*	*18*	*80*
**SGRQ scores**					
**symptoms**	24.2 (12.4–37.0)	50.8 (38.4–58.6)	46.7 (35.7–59.3)	46.5 (30.9–58.2)	67.9 (57.7–76.5)
**activity**	36.2 (24.1–47.7)	53.6 (47.7–66.2)	47.7 (35.6–62.6)	63.3 (54.4–69.1)	71.5 (59.5–82.7)
**impacts**	10.3 (5.5–16.9)	19.3 (13.9–31.4)	21.3 (11.7–36.6)	28.9 (18.3–46.7)	41.1 (24.9–59.7)
*N*	*188*	*94*	*132*	*17*	*80*
**CASIS total score**					
	5.7 (0.0–11.4)	10.0 (5.7–17.1)	20.0 (11.4–31.4)	28.6 (11.4–37.1)	40.0 (25.7–48.6)
*N*	*178*	*92*	*123*	*17*	*74*
**HADS scores**					
**Total**	5.0 (2.0–9.0)	9.0 (6.0–15.0)	8.0 (4.0–14.0)	9.0 (7.0–14.0)	16.0 (12.0–21.0)
**Anxiety**	2.0 (1.0–5.0)	5.0 (2.0–8.0)	4.0 (1.0–7.0)	5.0 (3.0–8.0)	8.0 (6.0–10.0)
**Depression**	2.0 (1.0–4.0)	5.0 (2.0–8.0)	4.0 (2.0–8.0)	4.0 (3.0–6.0)	8.0 (5.0–10.0)
**N**	*66*	*35*	*38*	*4*	*29*
**IPAQ–level of physical activity (n;%)**					
**Low**	14 (21.2)	8 (22.9)	10 (26.3)	2 (50.0)	13 (44.8)
**Moderate**	50 (75.8)	27 (77.1)	28 (73.7)	2 (50.0)	16 (55.2)
**High**	2 (3.0)	0 (0.0)	0 (0.0)	0 (0.0)	0 (0.0)

If not otherwise specified the median (25^th^ - 75^th^ percentile) were showed.

MILD COPD: No night-time symptoms.

MILD EMPHYSEMATOUS: Prevalent dyspnea in the early-morning and day-time, but not by night.

SEVERE BRONCHITIC: Nocturnal and diurnal cough and phlegm, no nocturnal dyspnea.

SEVERE EMPHYSEMATOUS: Nocturnal and diurnal dyspnea.

SEVERE MIXED COPD: Higher frequency of symptoms during 24h.

The patients in MILD COPD m-phenotype had better quality of sleep than patients of other m-phenotypes (p-values < .0001) (Table 4*)*. A negative grading of quality of sleep was evident from MILD COPD to MILD EMPHYSEMATOUS and, then, SEVERE BRONCHITIC, SEVERE EMPHYSEMATOUS and SEVERE MIXED COPD.

The patients with MILD COPD had lower HADS total, anxiety and depression scores than MILD EMPHYSEMATOUS or SEVERE MIXED COPD patients (p-values < .0001) and had lower total and depression scores than SEVERE BRONCHITIC patients (p-values < .0001) (Table 4*)*. Differences between HADS scores of patients with SEVERE MIXED COPD vs MILD EMPHYSEMATOUS or SEVERE BRONCHITIC patients were also significant (p-values < .0001).

Low physical activity, as expressed by the IPAQ score, largely prevailed in the patients with SEVERE MIXED COPD and SEVERE EMPHYSEMATOUS m-phenotypes. However, differences between m-phenotypes did not reach significance (Table 4*)*.

### M-phenotypes vs pharmacological therapies for COPD

Pharmacological therapy did not differ significantly between m-phenotypes (Table 5).

**Table 5:** Multidimensional phenotype vs pharmacological therapies for COPD at enrollment.

	MILD COPD	MILD EMPHYSEMATOUS	SEVERE BRONCHITIC	SEVERE EMPHYSEMATOUS	SEVERE MIXED COPD
	(N = 190)	(N = 94)	(N = 132)	(N = 18)	(N = 80)
COPD pharmacological therapy ongoing at enrollment (N; %)					
No therapy	25 (13.2)	17 (18.1)	20 (15.2)	1 (5.6)	8 (10.0)
Triple therapy	45 (23.7)	28 (29.8)	51 (38.6)	3 (16.7)	28 (35.0)
LABA+LAMA	48 (25.3)	23 (24.5)	25 (18.9)	6 (33.3)	22 (27.5)
LAMA Alone	38 (20.0)	12 (12.8)	13 (9.8)	2 (11.1)	12 (15.0)
ICS+LABA	26 (13.7)	7 (7.4)	19 (14.4)	4 (22.2)	8 (10.0)
OTHER	8 (4.2)	7 (7.4)	4 (3.0)	2 (11.1)	2 (2.5)

Triple therapy is defined as any combination of long-acting beta-agonists (LABA), long-acting muscarinic antagonist (LAMA), inhaled corticosteroid (ICS) in fixed dose combination or not.

Other therapies include LABA alone, ICS+LAMA, SABA, SAMA, Theophylline, Oral or IV Corticosteroids.

Percentages computed within m-phenotypes.

MILD COPD: No night-time symptoms.

MILD EMPHYSEMATOUS: Prevalent dyspnea in the early-morning and day-time, but not by night.

SEVERE BRONCHITIC: Nocturnal and diurnal cough and phlegm, no nocturnal dyspnea.

SEVERE EMPHYSEMATOUS: Nocturnal and diurnal dyspnea.

SEVERE MIXED COPD: Higher frequency of symptoms during 24h.

## Discussion

We found that the circadian rhythm of the three main respiratory symptoms and an index of disease-related health status could classify the vast majority of patients, whereas comorbidity, FEV1, age and gender added little to the classificatory power of the model. Indeed, based on these components of the latent factors, it was possible to identify five multidimensional phenotypes with distinctive pattern of disease (Fig 2): the MILD COPD, the MILD EMPHYSEMATOUS, the SEVERE BRONCHITIC, the SEVERE EMPHYSEMATOUS and the SEVERE MIXED COPD.

Taken together, these findings point out that the “core business” of COPD, the clinical one, can distinguish patients with different disease profiles. The fact that two factors, extracted through a parsimonious model, could explain 82% of intrinsic variability testifies to the strength of the classificatory procedure and to the overall quality of the clustering based mainly on clinical ground.

Consistent with the classical distinction of bronchitic and emphysematous phenotypes, dyspnea and productive cough contributed to define two well distinguished dimensions of the disease. Accordingly, attempts at optimally caring the individual COPD patient should have individually tailored goals: relieving bronchial obstruction might not be the main objective of the care in patients burdened mainly or exclusively with productive cough, whereas relieving dyspnea could be the core therapeutic goal in another group of patients, and the timing of symptoms further adds to the decision making [19]. This seemingly obvious finding has important practical implications. Indeed, FEV1 or the six minute walked distance should not be considered universal effect measures in randomized clinical trials. While these indexes have great merit as overall indexes of disease severity, they might not catch the core expression of COPD and, then, the main expected benefit of therapy in an important proportion of patients. The findings of the current study strongly advocate for the design of multi-outcome or patient-tailored RCTs in the perspective of precision medicine. Pursuing the patient centered objective of care is also expected to increase adherence to therapy. Indeed, the surprisingly high rate of non-adherence to inhaled medication, including inappropriate inhaler use by COPD patients [20] as well as the usually high rate of enrollees lost in RCT due to withdrawn consent [21–22] might reflect the perception of the care as missing the own needs by many patients.

The circadian rhythm of symptoms played a major classificatory role. Indeed, MILD COPD and MILD EMPHYSEMATOUS patients never/very unfrequently experience nighttime symptoms, which, instead, are always present in SEVERE EMPHYSEMATOUS, SEVERE BRONCHITIC and SEVERE MIXED COPD m-phenotypes. Several evidences support the role of circadian rhythm of symptoms in defining the clinical spectrum of COPD. Indeed, bronchial obstruction is strictly related to nocturnal symptoms [3] and wheezing has been reported to be the most common of them [23–24]. Interestingly, night time symptoms qualified as the main correlate of depression in the Assess study [5]. However, Physicians significantly underestimate the impact of COPD on the patient’s ability to get up in the morning and on sleep [6]. Unfortunately, only in the last few years attention has been paid to nocturnal symptoms, but mainly to empirically modulate the pharmacological therapy rather than to rigorously understand COPD heterogeneity.

Selected innovative models have recently been proposed to classify COPD patients. They variably rely upon comorbidities and complications of COPD [25–26] or on a comprehensive respiratory function assessment [27]. However, they do not rate symptoms according to a standardized procedure. This likely explains why symptoms played a major classificatory role in ours and not in these models. Thus, two different phylosophies, respectively centered on the non respiratory and the respiratory dimension of COPD, found previous model and the present one. Only the longitudinal observation will allow compare discriminative and prognostic properties of these models. On the other hand, classificatory models including analytes such as cytokines or genetic markers are intended to define idyotypes and not phenotypes and, thus, are not comparable with our prettily clinical and, then, phenotypic model [28]. Also the Spanish clinical classification of COPD patients differs from ours because it simply aims at assessing the perceived prevalence of predefined clinical phenotypes [29]. Given patients were on regular topical therapy for COPD, the important prevalence of night time symptoms and severely impaired health status testifies to the poor quality of the overall pharmacological approach. Analogously, in a European survey from primary care centers two thirds of COPD patients complained of dyspnea despite current treatment [30]; an even higher percentage of subjects suffered from chronic cough, and this was similar across all severity COPD stages. Otherwise, the important clinical differences among clusters suggest that many patients are expected to gain the greatest benefit from non- pharmacological measures contrasting phlegm and mucus. Indeed, being recognized as a multidimensional and heterogeneous condition, COPD should be the object of personalized care.

The fact that the SGRQ contributed to categorize COPD patients further confirms that health status is something different from and, thus, may not be merely expressed by symptoms. Interestingly, dyspnea associates with SGRQ in latent factor 2, as if dyspnea were the main correlate of perceived health status. This partly reflects the structure of the SGRQ, which largely relates the perceived status to physical activities which are obviously limited by dyspnea. Furthermore, this finding stresses the need of including an index of health status among the instruments rating COPD severity and the effects of therapeutic interventions.

Limitations of the study deserve consideration. First, we chose the classificatory variables with the aim of limiting recall bias. This is the reason why we selected the *Night-time*, *Morning and Day-time Symptoms of COPD questionnaire*, with a recall period of a week. Indeed, we based the classification on variables reflecting the patient’s condition at the time of enrollment in order to prevent any classificatory bias linked to uncertain definition of variables requiring an accurate recall, such as frequency of exacerbations. Second, the definition of a clinical variant or subtype of COPD should be founded on the clinical pattern in the absence of any therapy. However, it is almost impossible to recruit patients with severe COPD free from pharmacological treatment, and those untreated likely represent a poorly representative sample. Third, the observed clinical variants of COPD should not be considered true phenotypes. Instead, phenotypes are intrinsically stable, and only the follow-up will assess cluster stability. Thus, the full spectrum of classificatory properties of our model will emerge only at the end of the follow-up phase of the study, which is currently ongoing. Finally, our patients had mean age of 71.4 years; thus, present results might not fully apply to the growing fraction of COPD patients classified as old (age over 75 years), which pose distinctive diagnostic and therapeutic problems [31].

## Conclusions

Despite these limitations, our study emphasizes the clinical meaning of the circadian rhythm of COPD symptoms. It suggests that a classificatory approach based on the respiratory clinical dimension of COPD succeeds in identifying classes of patients with distinctive features. In an era characterized by a growing interest in extra pulmonary features of COPD, which obviously are worthy of consideration, our study redirects the attention of physicians to the elementary truth that COPD is a primarily respiratory disease and an accurate clinical rating of patient’s status is the first task required to any physician caring for a COPD patient.

## Supporting information

S1 Table Factor analysis output (eigenvalues of the reduced correlation matrix). This table shows a part of the output of factor analysis (eigenvalues of the reduced correlation matrix).
(DOC)

S2 Table Cluster analysis output (cluster history). This table shows a part of the output of cluster analysis (cluster history).
(DOC)

S1 Fig Factor analysis output (scatter plot of eigenvalues). This figure shows a part of the output of factor analysis (scatter plot of eigenvalues).
(DOC)

S2 Fig Cluster analysis output (dendrogram). This figure shows a part of the output of cluster analysis (dendrogram).
(DOC)

## References

[1] Lange P, Halpin DM, O'Donnell DE, MacNee W. Diagnosis, assessment, and phenotyping of COPD: beyond FEV1. Int J Chron Obstruct Pulmon Dis. 2016 2 19;11 Spec Iss:3–12. 26937185 10.2147/COPD.S85976 PMC4765947

[2] Kumar M, Seeger W, Voswinckel R. Senescence-associated secretory phenotype and its possible role in chronic obstructive pulmonary disease. Am J Respir Cell Mol Biol. 2014 9;51(3):323–33. 10.1165/rcmb.2013-0382PS 25171460

[3] Price D Proceedings of the IV REVIEW: NIGHT-TIME SYMPTOMS IN COPD A. AGUSTI ET AL. 192 VOLUME 20 NUMBER 121 EUROPEAN RESPIRATORY REVIEW World Asthma and COPD Forum (Paris, France, April 30 to May 3, 2011)

[4] Partridge MR, Karlsson N, Small IR. Patient insight into the impact of chronic obstructive pulmonary disease in the morning: an internet survey. Curr Med Res Opin. 2009 8;25(8):2043–8. 10.1185/03007990903103006 19569976

[5] Miravitlles M, Worth H, Soler Catalauña JJ, Price D, De Benedetto F, Roche N, et al Observational study to characterize 24-hour COPD symptoms and their relationship with patient-reported outcomes; results fromthe ASSESS study. Respir Res. 2014 10;21; 15: 122 10.1186/s12931-014-0122-1 25331383 PMC4220061

[6] Price D, Small M, Milligan G, Higgins V, Garcia Gil EG, Estruch J. Impact of night-time symptoms in COPD: a real-world study in five European countries. Int J Chron Obstruct Pulmon Dis. 2013; 8:595–603. 10.2147/COPD.S48570 24348032 PMC3849086

[7] Bellia V, Catalano F, Scichilone N, Incalzi RA, Spatafora M, Vergani C et al Sleep disorders in the elderly with and without chronic airflow obstruction: the SARA study. Sleep. 2003 5 1;26(3):318–23. 12749552 10.1093/sleep/26.3.318

[8] Canonica GW, Blasi F, Scichilone N, Simoni L, Zullo A, Giovannetti C et al on behalf of STORICO study group Characterization of circadian COPD symptoms by phenotype: Methodology of the STORICO observational study. Eur J Inter Med. 2017 9; 43: 62–68. 10.1016/j.ejim.2017.05.021 28576398

[9] Jones PW, Quirk FH, Baveystock CM, Littlejohns P. A self-complete measure for chronic airflow limitation. The St George's respiratory questionnaire. Am Rev Respir Dis. 1992 6;145 (6):1321–7. 1595997 10.1164/ajrccm/145.6.1321

[10] Meguro M, Barley EA, Spencer S, Jones PW. Development and validation of an improved COPD-specific version of the St George's respiratory questionnaire. Chest. 2007 8;132 (2):456–63. 17646240 10.1378/chest.06-0702

[11] Zigmond AS, Snaith RP. The hospital anxiety and depression scale. Acta Psychiatr Scand 1983;67:361–70. 6880820 10.1111/j.1600-0447.1983.tb09716.x

[12] Pokrzywinski RF, Meads DM, McKenna SP, Glendenning GA, Revicki DA. Development and psychometric assessment of the COPD and Asthma Sleep Impact Scale (CASIS). Health Qual Life Outcomes 2009;7:98 10.1186/1477-7525-7-98 19968881 PMC2794842

[13] Ainsworth BE, Bassett DR Jr, Strath SJ, Swartz AM, O'Brien WL, Thompson RW et al. Comparison of three methods for measuring the time spent in physical activity. Med Sci Sports Exerc 2000;32:S457–64. 10993415 10.1097/00005768-200009001-00004

[14] Miller MR, Hankinson J, Brusasco V, Burgos F, Casaburi R, Coates A et al Standardisation of spirometry. Eur Respir J 2005; 26: 319–338 16055882 10.1183/09031936.05.00034805

[15] Child D. (1990). The essentials of factor analysis, second edition London: Cassel Educational Limited.

[16] Guttman L. (1953) Image Theory for the Structure of Quantitative Variables, Psychometrica, 18, 277–296.

[17] Kaiser H.F., Rice J. Little Jiffy, Mark IV, Educational and Psychological Measurement. 1974; 34 (1), 111–117.

[18] Truong Y., McColl R. Intrinsic motivations, self-esteem, and luxury goods consumption. Journal of Retailing and Consumer Services.2011; 18 (6): 555–561

[19] Singh D, Miravitlles M, Vogelmeier C. Chronic Obstructive Pulmonary Disease Individualized Therapy: Tailored Approach to Symptom Management. Adv Ther. 2017 2;34(2):281–299. 10.1007/s12325-016-0459-6 27981495 PMC5331083

[20] Van Boven JFM, Lavorini F, Dekhuijzen PNR, Blasi F, Price DB, Viegi G Urging Europe to put non-adherence to inhaled respiratory medication higher on the policy agenda: a report from the First European Congress on Adherence to Therapy. Eur Respir J. 2017 5 19;49(5). 10.1183/13993003.00076-2017 28526801

[21] Calverley PM, Anderson JA, Celli B, Ferguson GT, Jenkins C, Jones PW et al Salmeterol and fluticasone propionate and survival in chronic obstructive pulmonary disease. N Engl J Med. 2007 2 22;356(8):775–89. 17314337 10.1056/NEJMoa063070

[22] Tashkin DP, Celli B, Senn S, Burkhart D, Kesten S, Menjoge S et al A 4-year trial of tiotropium in chronic obstructive pulmonary disease. N Engl J Med. 2008 10 9;359(15):1543–54. 10.1056/NEJMoa0805800 18836213

[23] Kessler R, Partridge MR, Miravitlles M, Cazzola M, Vogelmeier C, Leynaud D et al Symptom variability in patients with severe COPD: a pan-European cross-sectional study. Eur Respir J. 2011 2;37 (2):264–72. 10.1183/09031936.00051110 21115606

[24] Partridge MR, Karlsson N, Small IR. Patient insight into the impact of chronic obstructive pulmonary disease in the morning: an internet survey. Curr Med Res Opin. 2009 8;25 (8):2043–8. 10.1185/03007990903103006 19569976

[25] Burgel PR, Paillasseur J-L, Janssens W, Piquet J, ter Riet G, Garcia-Aymerich J et al A simple algorithm for the identification of clinical COPD phenotypes Eur Respir J 2017; 50: 1701034 10.1183/13993003.01034-2017 29097431

[26] Pikoula M, Quint JK, Nissen F, Hemingway H, Smeeth L, Denaxas S Identifying clinically important COPD sub-types using data-driven approaches in primary care population based electronic health records BMC Med Inform Decis Mak. 2019; 19: 86 10.1186/s12911-019-0805-0 30999919 PMC6472089

[27] Augustin IML, Spruit MA, Houben-Wilke S, Franssen FME, Vanfleteren LEGW, Gaffron S et al The respiratory physiome: Clustering based on a comprehensive lung function assessment in patients with COPD. PLoS One. 2018 9 12;13(9):e0201593 10.1371/journal.pone.0201593 30208035 PMC6135389

[28] Agusti A, Bel E, Thomas M, Vogelmeier C, Brusselle G, Holgate S et al Treatable traits: toward precision medicine of chronic airway diseases European Respiratory Journal 2016 47: 410–419; 10.1183/13993003.01359-2015 26828055

[29] Calle Rubio M, Casamor R, Miravitlles M. Identification and distribution of COPD phenotypes in clinical practice according to Spanish COPD Guidelines: the FENEPOC study. Int J Chron Obstruct Pulmon Dis. 2017 8 9;12:2373–2383. 10.2147/COPD.S137872 28848338 PMC5557116

[30] Jones PW, Brusselle G, Dal Negro RW, Ferrer M, Kardos P, Levy ML et al Health-related quality of life in patients by COPD severity within primary care in Europe. Respir Med. 2011 1;105(1):57–66. 10.1016/j.rmed.2010.09.004 20932736

[31] Corsonello A, Scarlata S, Pedone C, Bustacchini S, Fusco S, Zito A et al Treating COPD in Older and Oldest Old Patients. Curr Pharm Des. 2015;21(13):1672–89. 25633118 10.2174/1381612821666150130121229

