# Supplementary material for: The COPD multi-dimensional phenotype: A new classification from the STORICO Italian observational study
Source: PLoS One. 2019 Sep 13;14(9):e0221889. doi: 10.1371/journal.pone.0221889 (PMC6743765; doi:10.1371/journal.pone.0221889)
Supplement: S1 Table — This table shows a part of the output of factor analysis (eigenvalues of the reduced correlation matrix). (DOC) [file pone.0221889.s001.doc]

**Supplementary Data S1. Factor analysis output (eigenvalues of the reduced correlation matrix)**

| **Eigenvalues of the Reduced Correlation Matrix: Total = 7.80005982 Average = 0.33913304** | | | | |
| --- | --- | --- | --- | --- |
|  | **Eigenvalue** | **Difference** | **Proportion** | **Cumulative** |
| **1** | 4.56823587 | 2.76131971 | 0.5857 | 0.5857 |
| **2** | 1.80691616 | 1.06517227 | 0.2317 | 0.8173 |
| **3** | 0.74174389 | 0.10608441 | 0.0951 | 0.9124 |
| **4** | 0.63565948 | 0.07514733 | 0.0815 | 0.9939 |
| **5** | 0.56051215 | 0.19285072 | 0.0719 | 1.0658 |
| **6** | 0.36766143 | 0.09631648 | 0.0471 | 1.1129 |
| **7** | 0.27134495 | 0.06129995 | 0.0348 | 1.1477 |
| **8** | 0.21004500 | 0.09739428 | 0.0269 | 1.1746 |
| **9** | 0.11265073 | 0.03104133 | 0.0144 | 1.1891 |
| **10** | 0.08160939 | 0.01551264 | 0.0105 | 1.1995 |
| **11** | 0.06609675 | 0.07557487 | 0.0085 | 1.2080 |
| **12** | -.00947812 | 0.02226954 | -0.0012 | 1.2068 |
| **13** | -.03174766 | 0.01494331 | -0.0041 | 1.2027 |
| **14** | -.04669097 | 0.02870050 | -0.0060 | 1.1967 |
| **15** | -.07539146 | 0.02362819 | -0.0097 | 1.1871 |
| **16** | -.09901965 | 0.01533816 | -0.0127 | 1.1744 |
| **17** | -.11435782 | 0.04867813 | -0.0147 | 1.1597 |
| **18** | -.16303595 | 0.01112539 | -0.0209 | 1.1388 |
| **19** | -.17416134 | 0.02504927 | -0.0223 | 1.1165 |
| **20** | -.19921061 | 0.00545442 | -0.0255 | 1.0909 |
| **21** | -.20466503 | 0.03262981 | -0.0262 | 1.0647 |
| **22** | -.23729484 | 0.03006769 | -0.0304 | 1.0343 |
| **23** | -.26736253 |  | -0.0343 | 1.0000 |

| *3 factors will be retained by the NFACTOR criterion.* |
| --- |
