# Supplementary material for: The COPD multi-dimensional phenotype: A new classification from the STORICO Italian observational study
Source: PLoS One. 2019 Sep 13;14(9):e0221889. doi: 10.1371/journal.pone.0221889 (PMC6743765; doi:10.1371/journal.pone.0221889)
Supplement: S2 Table — This table shows a part of the output of cluster analysis (cluster history). (DOC) [file pone.0221889.s002.doc]

**Supplementary Data S3. Cluster analysis output (cluster history)**

| **Cluster History** | | | | | | | | | | | |
| --- | --- | --- | --- | --- | --- | --- | --- | --- | --- | --- | --- |
| **Number of Clusters** | **Clusters Joined** | | **Freq** | **Semipartial R-Square** | **R-Square** | **Approximate Expected R-Square** | **Cubic Clustering Criterion** | **Pseudo F Statistic** | **Pseudo t-Squared** | **Norm RMS Distance** | **Tie** |
| 30 | CL134 | CL94 | 60 | 0.0000 | 1.00 | .972 | . | . | . | 73E-18 | T |
| 29 | CL160 | CL47 | 12 | 0.0000 | 1.00 | .971 | . | . | . | 73E-18 | T |
| 28 | CL71 | CL42 | 8 | 0.0000 | 1.00 | .970 | . | . | . | 73E-18 | T |
| 27 | CL33 | CL68 | 23 | 0.0000 | 1.00 | .969 | . | . | . | 78E-18 | T |
| 26 | CL120 | CL56 | 29 | 0.0000 | 1.00 | .967 | . | . | . | 1E-16 | T |
| 25 | CL36 | CL28 | 44 | 0.0000 | 1.00 | .966 | . | . | . | 15E-17 | T |
| 24 | CL30 | CL45 | 62 | 0.0000 | 1.00 | .964 | . | . | . | 17E-17 |  |
| 23 | CL27 | CL38 | 30 | 0.0023 | .998 | .963 | 66.2 | 9718 | . | 0.3309 | T |
| 22 | CL32 | CL39 | 41 | 0.0027 | .995 | .961 | 48.7 | 4625 | . | 0.3309 | T |
| 21 | CL34 | CL40 | 65 | 0.0049 | .990 | .959 | 33.8 | 2449 | . | 0.3309 | T |
| 20 | CL25 | CL37 | 61 | 0.0052 | .985 | .957 | 25.1 | 1684 | . | 0.3309 | T |
| 19 | CL29 | CL50 | 16 | 0.0013 | .984 | .954 | 24.6 | 1641 | . | 0.3309 | T |
| 18 | CL24 | CL326 | 64 | 0.0008 | .983 | .951 | 24.9 | 1656 | . | 0.3309 | T |
| 17 | CL43 | CL41 | 51 | 0.0054 | .977 | .948 | 19.9 | 1337 | . | 0.3309 | T |
| 16 | CL417 | IT001-017 | 5 | 0.0003 | .977 | .945 | 21.2 | 1408 | . | 0.3309 | T |
| 15 | CL31 | CL106 | 50 | 0.0041 | .973 | .941 | 19.0 | 1277 | . | 0.3309 | T |
| 14 | CL35 | CL26 | 89 | 0.0083 | .965 | .936 | 14.3 | 1045 | . | 0.3309 | T |
| 13 | CL46 | CL59 | 15 | 0.0013 | .963 | .931 | 15.5 | 1094 | . | 0.3309 | T |
| 12 | CL16 | CL44 | 29 | 0.0018 | .961 | .925 | 16.5 | 1137 | 145 | 0.3625 |  |
| 11 | CL18 | CL19 | 80 | 0.0057 | .956 | .918 | 15.5 | 1085 | 212 | 0.3723 |  |
| 10 | CL22 | CL23 | 71 | 0.0074 | .948 | .909 | 14.3 | 1027 | 101 | 0.3827 |  |
| 9 | CL17 | CL14 | 140 | 0.0141 | .934 | .899 | 11.1 | 897 | 141 | 0.403 |  |
| 8 | CL10 | CL20 | 132 | 0.0349 | .899 | .886 | 3.35 | 646 | 257 | 0.5831 |  |
| 7 | CL13 | CL101 | 18 | 0.0032 | .896 | .869 | 6.29 | 729 | 41.0 | 0.592 |  |
| 6 | CL15 | CL9 | 190 | 0.0419 | .854 | .846 | 1.50 | 595 | 247 | 0.603 |  |
| 5 | CL21 | CL12 | 94 | 0.0260 | .828 | .814 | 2.22 | 613 | 337 | 0.6095 |  |
| 4 | CL6 | CL5 | 284 | 0.0842 | .744 | .767 | -2.9 | 494 | 222 | 0.7302 |  |
| 3 | CL11 | CL8 | 212 | 0.1859 | .558 | .688 | -12 | 323 | 646 | 1.0414 |  |
| 2 | CL4 | CL7 | 302 | 0.0703 | .488 | .502 | -1.0 | 488 | 108 | 1.1409 |  |
| 1 | CL2 | CL3 | 514 | 0.4878 | .000 | .000 | 0.00 | . | 488 | 1.2363 |  |
