# Supplementary material for: The COPD multi-dimensional phenotype: A new classification from the STORICO Italian observational study
Source: PLoS One. 2019 Sep 13;14(9):e0221889. doi: 10.1371/journal.pone.0221889 (PMC6743765; doi:10.1371/journal.pone.0221889)
Supplement: S1 Fig — This figure shows a part of the output of factor analysis (scatter plot of eigenvalues). (DOC) [file pone.0221889.s003.doc]

**Supplementary Data S2. Factor analysis output (scatter plot of eigenvalues)**

|  |
| --- |
| Scree Plot of Eigenvalues |
| | |
| | |
| | |
| 5.0 + |
| | |
| | |
| | 1 |
| 4.5 + |
| | |
| | |
| | |
| 4.0 + |
| | |
| | |
| | |
| 3.5 + |
| | |
| | |
| | |
| 3.0 + |
| E | |
| i | |
| g | |
| e 2.5 + |
| n | |
| v | |
| a | |
| l 2.0 + |
| u | |
| e | 2 |
| s | |
| 1.5 + |
| | |
| | |
| | |
| 1.0 + |
| | |
| | 3 |
| | 4 |
| 0.5 + 5 |
| | 6 |
| | 7 8 |
| | 9 0 1 |
| 0.0 + 2 3 4 |
| | 5 6 7 8 9 |
| | 0 1 2 3 |
| | |
| -0.5 + |
| | |
| | |
| | |
| -----+----+----+----+----+----+----+----+----+----+----+----+----+----+----+----+----+----+----+----+----+----+----+----+---- |
| 0 1 2 3 4 5 6 7 8 9 10 11 12 13 14 15 16 17 18 19 20 21 22 23 |
|  |
| Number |
