# Supplementary material for: The COPD multi-dimensional phenotype: A new classification from the STORICO Italian observational study
Source: PLoS One. 2019 Sep 13;14(9):e0221889. doi: 10.1371/journal.pone.0221889 (PMC6743765; doi:10.1371/journal.pone.0221889)
Supplement: S2 Fig — This figure shows a part of the output of cluster analysis (dendrogram). (DOC) [file pone.0221889.s004.doc]

**Supplementary Data S4. Cluster analysis output (dendrogram)**
